# Preterm Premature Rupture of Membranes in Human Immunodeficiency Virus-Infected Women: A Novel Case Series

**DOI:** 10.1155/IDOG/2006/53234

**Published:** 2006-04-20

**Authors:** Kjersti M. Aagaard-Tillery, Monique G. Lin, Virginia Lupo, Alan Buchbinder, Patrick S. Ramsey

**Affiliations:** ^1^Division of Maternal-Fetal Medicine, Department of Obstetrics and Gynecology, School of Medicine, The University of Utah Health Sciences, 30 North 1900 East, SOM 2B200, Salt Lake City, UT 84132-2209, USA; ^2^Division of Maternal-Fetal Medicine, Department of Obstetrics and Gynecology, Hennepin County Medical Center, Minneapolis, MN 55415 USA; ^3^Center for Research in Women's Health, The University of Alabama at Birmingham, Birmingham, AL 35294, USA

## Abstract

*Objective*. To evaluate the management and outcomes of a series of human immunodeficiency virus-(HIV-) infected women whose pregnancies were complicated by preterm premature rupture of membranes (PPROM). *Study design*. We conducted a retrospective chart review of all women with confirmed HIV infection who had a pregnancy complicated by PPROM remote from term. PPROM remote from term was defined as rupture of membranes prior to 32-week gestation. Collective cases from two centers (Hennepin County Medical Center and The University of Alabama at Birmingham) were reviewed and data on management and outcomes were abstracted. *Results*. Of the HIV-positive women, we identified 291 pregnancies having occurred in the study interval from two institutions. Of these pregnancies, 7 (2.4%) developed PPROM remote from term with subsequent delivery from 25- to 32-week gestation. Vertical HIV transmission was noted in 2 of 6 children whose long-term followup status was confirmed (33%) of these cases. However, both of these cases occurred in women with either no antepartum/intrapartum antiviral therapy or where only zidovudine monotherapy was used. Importantly, in spite of expectant management, no cases of vertical HIV transmission occurred in women who were receiving either multidrug or highly active antiviral therapy (HAART) at the time of PPROM and who had a cesarean delivery in cases where the predelivery viral load > 1000 copies/mL. *Conclusion*. Our limited observations raise the question as to whether in the current era of multidrug therapy immediate delivery should be undertaken in HIV+ pregnancies complicated by PPROM at an early gestational age. This case series further suggests that in those pregnancies that lend themselves to expectant management, such a strategy may be considered appropriate.

## INTRODUCTION

Preterm premature rupture of membranes (PPROM) occurs in approximately 3% of all pregnancies, accounting for nearly 1/3 of all preterm births [1–5] . Moreover, PPROM is a contributor to perinatal morbidity and mortality—primarily due to its association with delivery remote from term, chorioamnionitis, placental abruption, and umbilical cord compression or prolapse [1, 2]

Management of women with HIV infection who develop PPROM poses an obstetric dilemma. Limited data are available to direct optimal management of HIV-positive women who develop PPROM. Previous investigations have demonstrated that in term pregnancies complicated by maternal HIV infection, duration of membrane rupture > 4 hours increases the risk of intrapartum vertical transmission [12–17]. By extrapolation of this data, some authors suggest that women with HIV who undergo PPROM should not be managed conservatively [1, 12–14]. However, in instances near the limits of viability, conservative management of PPROM may be justified because of the extremely high risk of mortality and chronic morbidity when immediate delivery is prompted [1]. We and others [1] have been unable to identify any published manuscripts to date reporting on conservative management of PPROM in the setting of HIV (Medline search 1 January 1966–1 September 2005: search terms: preterm premature rupture of membranes, human immunodeficiency virus infection, acquired immunodeficiency syndrome). Given an absolute lack of published reports, we sought to provide an initial report on the management and outcomes of a series of HIV-infected women whose pregnancies were complicated by preterm premature rupture of membranes (PPROM).

## MATERIALS AND METHODS

*Discussion of management options*

In each instance, a thorough and complete review of the relative risks of prematurity and PPROM remote from term and potential for vertical transmission with prolonged rupture of the membranes was reviewed with the patient, and documented in the patient chart. These discussions involved obstetrician/gynecologists, maternal-fetal medicine specialists, neonatologists, and/or infectious disease specialists, with language interpreters as indicated.

*Study population*

At Hennepin County Medical Center (HCMC), the HIV-infected women were identified through the HCMC HIV database encompassing the interval from 1990 to 2004, and the electronic medical records (EMRs) system was reviewed to identify whether they had been seen through the obstetrics and gynecology clinics, high-risk obstetrics clinics, or on labor and delivery. For those with identified encounters, exhaustive review of both the EMR and patient chart was undertaken to ascertain whether a pregnancy had occurred, and whether the pregnancy had been complicated by PPROM remote from term. At The University of Alabama at Birmingham (UAB), an electronic obstetric database (OBAR) of women who obtained their prenatal care and delivered at UAB from 1980 to 2004 was searched. Women with HIV having undergone PROM were identified and for those women in whom latency was greater than 24 hours, an exhaustive chart review was performed.

*Definitions*

PPROM remote from term was defined as preterm, premature rupture of the membranes prior to 32-week gestation confirmed by ferning, pooling, or indigo-carmine amnioinfusion; dating was by previously published American College of Obstetricians and Gynecologist (ACOG) criteria and employed last menstrual period with fetal biometry by ultrasonographic determination [22]. Maternal HIV-positive status was defined as documentation of HIV-1 or HIV-2 seroconversion, confirmed by Western blot analysis. AIDS was defined as an absolute CD4+, CD3+ lymphocyte count less than 200 ×10^6^ cells/L. Infants were considered infected if they were HIV-seropositive, confirmed by Western blot at > 18 months of age, had two or more positive PCR tests or viral cultures at any age, had an AIDS-defining illness, or if they died with an HIV-related condition.

*Data extraction*

Once a pregnancy was identified as having been dually complicated by HIV-positive maternal status and PROM remote from term, data was uniformly extracted from the patients chart with respect to maternal characteristics (age, ethnicity, parity, year of HIV-positive diagnosis, past medical history, polysubstance abuse history, and infectious comorbidities), prenatal care (gestational age and dating criteria, gestational age at initiation of prenatal care, gestational age at initiation of HAART/therapy, number of prenatal visits), HIV characteristics (genotype, AIDS, infectious comorbidities [GBS, *trichomonas, gonorrhea, Chlamydia*, PPD, Hepatitis A, B, or C]), PPROM characteristics (means of confirmation, most recent viral load and CD4 count prior to PPROM, antiviral therapy at PPROM, gestational age at delivery, latency interval, indication for delivery, clinical or histologic evidence of chorioamnionitis), use of antiviral and antimicrobial therapy (HAART/intrapartum AZT, duration of use, utilization of antibiotics for the prolongation of latency), maternal peripartum complications (sepsis, chorioamnionitis, endometritis), and fetal and infant characteristics (administration of steroids for the promotion of fetal lung maturity, infant sex, birth weight, APGARS, cord gasses, NICU admission and duration of NICU stay, neonatal sepsis, RDS, IVH, necrotizing enterocolitis, vertical transmission, other complications or medical morbidities).

*IRB approval*

At HCMC, the HIV database for women with HIV is supported by Infectious Disease HIV Clinic with IRB approval. As this represented a case series, no further IRB approval was deemed necessary. At UAB, expedited IRB approval was obtained for chart review and data extraction.

## RESULTS

*Patient characteristics (Table 1)*

Over the indicated study intervals at HCMC and UAB, Birmingham, we identified 291 pregnancies having occurred in the cohort of HIV-positive women. Of these pregnancies, 7 (2.4%) developed PPROM with subsequent delivery from 25-to 32-week gestation. With the exception of one patient (patient 1), all women received prenatal care in their first or early midtrimester. For those who received adequate prenatal care, medical comorbidities reported reflect those found in the at-large HCMC and UAB populations.

*HIV and infectious morbidity (Table 2)*

With the exception of two patients (patients 2 and 6), all women were aware of their diagnosis of HIV prior to the pregnancy. Note that the diagnosis of HIV seroconversion (for patient 1) occurred in a prior pregnancy, but treatment was never initiated secondary to failed followup; this was in spite of multiple attempts to encourage compliance with care.

Each of the patients who received prenatal care at HCMC was initiated on HAART in the early midtrimester; two of these patients continued a modification of their prepregnancy therapy (Table 2). At UAB, both pregnancies occurred (1993) prior to the use of HAART in pregnancy. One woman (patient 6) was part of the PACTG 076 trial and was randomized to placebo. The second patient was receiving AZT prepregnancy and continued this regimen antepartum.

All patients were screened for viral and bacterial infectious comorbidities on initial presentation; all were rescreened on admission. None were Hepatitis B surface antigen-positive, one was Hepatitis C positive (patient 7); one (patient 3) had been previously exposed to Hepatitis A but was without active disease. Three of the 7 patients had *Trichomonas vaginalis* detected and treated, and one woman (patient 1) was positive and treated for *Trichomonas, C trachomatis*, and *N gonorrhea* at the time of admission. All women were RPR nonreactive on admission (data not shown). Two women (patients 3 and 7) had a history of tuberculosis, and had previously received therapy accordingly.

*PPROM and delivery characteristics (Table 3)*

Each patient in our series was distinct from the others with respect to gestational age at the time of PPROM; however, the overall profile typifies that seen among HIV-negative women with PPROM. In one instance (patient 2), PPROM occurred prior to viability and extensive counseling regarding the risks of oligohydramnios and prolonged ruptured membranes was undertaken. The patient elected to pursue expectant management.

With respect to viral load at the time of PPROM, one woman (patient 1) was hospitalized for preterm labor when rupture of membranes occurred. With respect to patients 2 and 5, noncompliance with outpatient HAART was problematic prior to PPROM. However, with inpatient management (patient 5) and frequent outpatient visits (patient 2), compliance improved and the viral load declined accordingly (Table 3). As per protocol standard at the time of presentation, viral loads were not obtained in the women that delivered at UAB.

All patients with ongoing pregnancies at HCMC received intravenous AZT for a maximum of 48 hours following membrane rupture, with repeat administration of therapy proximal to delivery. During the same interval, HAART was utilized as per their antepartum regimen (Table 3). The single exception to optimal utilization of intrapartum AZT use was patient 3, as she had experienced a prior episode of neutropenic sepsis with ARDS. At UAB, intrapartum AZT was not administered to one woman (patient 6) as per study (PACTG 076) protocol following randomization to placebo. In another patient, intravenous AZT was administered for 60 hours during her prolonged latent phase (from the time of admission to the time of delivery).

The mean latency in this cohort was 17.1 days with a median of 5 days; one woman (patient 2) had a latency of 92 days. Indications for delivery typified that of pregnancies complicated by PPROM, and mode of delivery was determined by obstetrical indications (Table 3).

*Maternal complications (Table 3)*

Clinical or histologic evidence of chorioamnionitis was prevalent; endometritis ensued in four cases. There was one instance of maternal sepsis with positive blood cultures for *Peptostreptococcus*. Otherwise, the duration of postdelivery hospitalization mirrored that of the general obstetric population at the respective institutions.

*Infant sequelae and complications (Table 4)*

Neonatal outcomes of the pregnancies in our series are shown in Table 4. Vertical HIV transmission was noted in 2 of the possible 6 cases (33%). In the two instances of vertical transmission, one woman and infant pair received no antiretroviral therapy as per study (PACTG 076) protocol. In the second instance, AZT was the only antiretroviral employed and viral loads were not used to guide delivery practice. All infants (except for patient 6 (PACTG 076 protocol)) received antiretroviral therapy in the neonatal period as per standard of care. Of the cases without vertical transmission in our series, all women received HAART at the time of PPROM with cesarean delivery for those with a viral load > 1000 copies/mL.

## COMMENTS

In our series, there were no instances of vertical transmission in women with PPROM who were receiving multidrug therapy or HAART despite prolonged intervals of rupture of membranes. The only two cases of vertical transmission observed were in one woman who did not receive antepartum or intrapartum antiretroviral therapy, and in another who received only AZT in the antepartum period. Our observations suggest that in the era of HAART, one might consider whether or not immediate delivery should be undertaken in pregnancies complicated by PPROM at an early gestational age. Alternatively, in pregnancies that may lend themselves to expectant management, such a strategy should be entertained.

In pregnancies complicated by maternal HIV infection, duration of rupture of membranes > 4 hours, in term pregnancies appear to increase the risk of intrapartum vertical transmission [12–17]. In one study [19], prolonged rupture of membranes in term (> 37 weeks) relative to preterm (< 37 weeks) was assessed. The authors found a 3.8-fold RR (95% CI 1.9, 7.8) increase in intrapartum HIV transmission when comparing preterm to term infants whose membranes had been ruptured > 4 hours. These authors attempted to delineate whether vertical transmission occurred in the intrauterine or intrapartum period and came to the conclusion that “preterm infants are at increased risk of acquiring infection at the time of delivery,” see [19]. However, transmission risk was not stratified by use of antiretroviral therapy, gestational age, nor mode of delivery [19]. As with this study, other studies [12–17] exploring the relationship of duration of membrane rupture and perinatal HIV transmission have failed to account for the use of HAART and viral load at the time of delivery.

One recent analysis [23] investigated the risk of perinatal HIV transmission in women on combination antiretroviral therapy with viral loads < 1000 copies/mL and membrane rupture > 4 hours. Duration of membrane rupture was not a risk factor for vertical transmission. Interestingly, monotherapy (OR 6.3, CI 2.4−16.6) or lack of antiretroviral therapy (OR 17.4 CI 6.7−45.5) and viral load > 1000 copies/mL (OR 9.9, CI 1.2−84.5) were independently associated with neonatal infection. Our case series suggests that this relationship between combination antiretroviral therapy and reduction in the rate of vertical transmission may apply to pregnancies complicated by prolonged premature preterm rupture of membranes and HIV.

We recognize that the primary limitation of this study is its limited sample size and its heterogeneous population. Our series does not claim to provide any statistical risk assessment for counseling patients on the relative merits or risks of transmission in the setting of PPROM remote from term. Rather, it is our hope that this novel series will provide an impetus to further reports and larger multi-institutional investigations to address this important clinical issue.

## CONDENSATION

We report a novel case series describing management and outcomes in human immunodeficiency virus-infected women who developed preterm premature rupture of membranes remote from term.

## Figures and Tables

**Table 1:** Patient characteristics on admission. PNC: prenatal care; ARDS: adult respiratory distress syndrome; AZT: zidovudine; AIDS: acquired immunodeficiency syndrome; N/A: data not available.

Patient	Age (years)	Parity	Ethnicity	Gestational age	Medical comorbidities	Substance abuse	HIV diagnosis in pregnancy	Duration of	AIDS
				at initiation of				HIV infection	
				prenatal care				(years)	

1	24	2103	African	No PNC prior	Polysubstance	Yes	No	1	No
			American	to admission	abuse	(cocaine)			

2	25	1001	Caucasian	13 0/7	Polysubstance	Yes	Yes	0	No
					abuse	(cocaine)			

3	30	5013	African	15 3/7	History of	No	No	3	No
					neutropenic sepsis				
					with ARDS				
					secondary to AZT				
					therapy				

4	30	1001	Latino	5 2/7	Chronic pain	No	No	11	No
					Anxiety				
					Appendicitis				
					(21-week gestation)				

5	33	2012	Native	7 0/7	Remote history of	Yes	No	7	No
			American		pyelonephritis	(marijuana alcohol)			

6	19	0211	African-American	9 2/7	None	No	Yes	0	No

7	29	1021	African-American	7 2/7	Laparotomy		No	0	No
					(trauma),	Yes			
					appendectomy,	(cocaine,			
					pneumonia,	alcohol,			
					Hepatitis C, history	tobacco)			
					of syphilis				

**Table 2:** HIV and infectious morbidity. HIV: human immunodeficiency virus; AIDS: acquired immunodeficiency syndrome; HAART: highly active antiretroviral therapy; AZT: zidovudine; INH: isoniazide; PACTG: the Pediatric AIDS Clinical Trials Group; TB: tuberculosis; S/P: status-post.

Patient	Prepregnancy therapy	Initial viral load (copies/mL)	Initial CD4 count (abs/mm^3^)	Gestational	Antepartum therapy	Vaginal	Viral coinfections	PPD status
				age at		infections		
				initiations		in this		
				of HAART		pregnancy		

1	None	2672	508	None	AZT	*Trichomonas*	None	Negative
						*C trachomatis*		
						*Gonorrhea*		

2	None	179	858	15 0/7	Combivir	None	None	Negative
					Neviripine			

3	Neviripine	< 50	684	15 3/7	Neviripine	None	Hepatitis A	Positive, s/p
	Lamivudine				Lamivudine			INH
	Stavudine				Stavudine			prophylaxis

4	Combivir	< 50	407	13 0/7	Combivir	None	None	Negative
					Neviripine			

5	None	971	312	14 0/7	Combivir	*Trichomonas*	None	Negative

6	None	N/A	766	None	(Placebo	None	None	Negative
					arm of			
					PACTG			
					076)			

7	AZT	N/A	N/A	None	AZT	*Trichomonas*	Hepatitis C	h/o TB, s/p
								therapy

**Table 3:** PPROM and delivery characteristics. PPROM: preterm premature rupture of membranes; HAART: highly active antiretroviral therapy; AZT: zidovudine; C/S: cesarean delivery; IUFD: intrauterine fetal demise; NSVD: spontaneous vaginal delivery; N/A: data not available.

Patient	Gestational	Viral load at	CD4 count	HAART at PPROM	Intrapartum	Latency antibiotics	Interval to	Viral load at	Indication for delivery	Mode of delivery	Clinical	Histologic	Histologic funisitis
	age at	PPROM	at PPROM		AZT therapy		delivery	delivery			chorio	chorio	
	PPROM	(copies/mL)	(abs/mm^3^)		(days)		(days)	(copies/mL)			amnionitis	amnionits	

1	30 5/7	1809	508	Combivir	Yes	No	1	1809	Labor prior classical	C/S	No	No	No
					(2 hours)								

2	17 2/7	7319	724	None	None	No	92	179	IUFD	SVD	No	Unknown	Unknown
					indicated								

3	30 2/7	< 50	684	Neviripine Lamivudine Stavudine	No (adverse reaction)	Yes	5	< 50	Chorio nonreassuring fetal status	C/S	Yes	Yes	Yes

4	31 4/7	< 50	407	Combivir	Yes	Yes	2	< 50	Chorio	SVD	Yes	Yes	Yes
				Neviripine	(2 days)								

5	24 2/7	3710	312	Combivir	Yes	No	10	971	Labor	C/S	No	Yes	Yes
					AZT (2 days);				nonreassur-				
					HAART (8 days);				ing fetal				
					AZT (4 hours)				status				

6	31 0/7	N/A	459	None	None	Yes	3	N/A	Chorio	SVD	Yes	N/A	N/A

7	23 4/7	N/A	N/A	None	Yes	Yes	7	N/A	Labor	SVD	No	N/A	N/A
					(3 days)								

**Table 4:** Infant sequelae and complications. N/A: not assessable, secondary to intrauterine fetal death. NICU: neonatal intensive care unit; RDS: respiratory distress syndrome; IVH: intraventricular hemorrhage; NEC: necrotizing enterocolitis; IUFD: intrauterine fetal demise; VSD: ventriculoseptal defect.

Patient	Delivery	Infant gender	Birth weight (g)	Apgars (1,5 m)	NICU	Antenatal steroids	Neonatal complications	Vertical transmission
	gestational				admission			
	age (weeks)				(days)			

1	31 5/7	Male	1660	6, 9	29	Yes	RDS	No

2	30 2/7	Female	710	0, 0	IUFD	Yes	IUFD	N/A

3	31 0/7	Male	1360	6, 7	53	Yes	None	No

4	31 6/7	Female	2000	9, 9	7	Yes	None	No

5	25 5/7	Male	778	3, 3	148	Yes	RDS, pyloric	No
							stenosis, VSD	
							retinopathy of	
							prematurity,	
							fetal alcohol	
							syndrome	

6	31 3/7	Male	1605	5, 8	29	No	RDS,	Yes
							neutropenia	

7	24 4/7	Male	827	1, 7	60	No	RDS,	Yes
							IVH grade 1, anemia	
